# The Identified Skeletal Collection of the School of Legal Medicine: a contemporary osteological collection housed in Universidad Complutense de Madrid, Spain

**DOI:** 10.1007/s00414-023-03047-5

**Published:** 2023-06-29

**Authors:** Catherine Villoria Rojas, Pilar Mata Tutor, Elena Labajo González, Bernardo Perea Pérez, Andrés Santiago Sáez, María García Velasco, Cindy Mansour, María Benito Sánchez

**Affiliations:** Department of Legal Medicine, Psychiatry and Pathology - Forensic Anthropology Laboratory. Avd. Complutense sn, Facultad de Medicina. Pabellón VII, Planta 3, Despacho, 111 Madrid, Spain

**Keywords:** Forensic anthropology, Population data, Identified skeletal collection, XXI century, The Spanish population

## Abstract

Osteological collections are an important resource for the development of methods to assist in the study of skeletal remains in archeological and/or forensic contexts. The aim is to describe the current characteristics of the Identified Skeletal Collection of the School of Legal Medicine and its historical context. The Identified Skeletal Collection of the School of Legal Medicine of the Complutense University of Madrid consists of 138 male and 95 female individuals, born between 1880 and 1980 and deceased between 1970 and 2009. The minimum age of the sample is perinatal and the maximum age is 97 years. The collection is an essential tool for forensic research, given that its population characteristics can be extrapolated to those of present-day Spain. Access to this collection offers unique teaching opportunities as well as provides the information necessary to develop various lines of research.

## Introduction

Contemporary osteological collections are the main resources used by physical and forensic anthropologists to develop tools to assist in the study of skeletal remains in archeological and/or forensic contexts, based on population-specific methodologies [1, 2]. The information gathered from these collections reflects the phenotypic variability of the region and makes it possible to refine methods that use biological characteristics to discriminate between individuals [3, 4]. These collections, in turn, facilitate comparative paleopathology studies, broaden knowledge of trauma types by analyzing their macroscopic characteristics, and analyze the influence of taphonomic phenomena on the human body [5, 6]. In addition, the study of large groups of contemporary individuals provides relevant information regarding biological and anatomical changes that reflect migration, feeding, and demographic patterns that can be used in archeological studies to make comparisons between populations and even between individuals from different periods in the same country [7, 8].

Due to intra-population variability, age-related changes and sexual maturation rates are usually specific to each population, which makes it necessary to construct demographic and epidemiological profiles of identified individuals to develop a specific methodology [9]. Knowledge of the phenotypic variability of a particular population increases the accuracy of identification processes in forensic contexts [10, 11]. In addition, knowing the cause of death of the individuals, as is the case in this collection, can also be a useful tool for the study of the pattern of lesions associated with specific pathologies and/or trauma presented in each situation. For these reasons, various osteological collections have been established throughout the 20th century all over the world. Within the Iberian Peninsula, there are seven well-established collections of identified contemporary skeletons: four in Portugal [3, 7, 12–15] and three in Spain [8, 16, 17]. All the collections, except the collection of the University of Granada, which has fetal and infant individuals up to 8 years of age [16], are made up of individuals ranging from 0 to 104 years of age, which has helped in the study of individuals of unknown origin of different sexes and ages [18, 19] (Table 1).

**Table 1:** Existing identified skeletal collections in the Iberian Peninsula

Collection	Reference	Country	Individuals (*N*)	Sex (*N*)		Age at death	Date of death
				♂	♀		
Bocage Museum Identified Skeletal Collection	Cardoso 2006	Portugal	1692	326	373	Birth–98	1880–1975
The Coimbra Identified Skeletal Collection	Cameriere 2007 & Hens 2008	Portugal	505	266	239	6–109	1904–1938
Universitat Autonoma of Barcelona Identified Skeletal Collection	Rissech 2011	Spain	35	19	16	31–97	1977–1991
Granada Osteological Collection of Identified Infants and Young Children	Alemán 2012	Spain	230	128	93	Prenatal–8	1970–2009
21st Century Identified Skeletal Collection	Ferreira 2014	Portugal	159	73	85	29–99	1995–2008
The Identified Skeleton Collection of Évora	Lopes 2021	Portugal	208	103	105	3–95	1870–1993
The Identified Skeletal Collection of the School of Legal Medicine		Spain	238	138	95	1–97	

The osteological collection of the School of Forensic Medicine of the Complutense University of Madrid has been mentioned in several scientific publications [2, 11, 12, 20], doctoral theses [17, 21, 22], and is included in the FASE database (http://forensicanthropology.eu/osteological-collections/#page-content) [23]. However, the available information is outdated. For this reason, this study is aims at describing the current characteristics of the Identified Skeletal Collection of the School of Legal Medicine and its historical context to raise awareness of the collection among the scientific community and researchers who may be interested in developing research projects.

## The Identified Skeletal Collection of the School of Legal Medicine

The exhumations were obtained thanks to an agreement between the Complutense University of Madrid and the Funeral Services of the Community of Madrid, which has been maintained until the present day. Exhumations of unclaimed human remains are usually conducted between 10 and 11 years after the death of the individual by the Law on Mortuary Health of the Autonomous Community of Madrid [24]. Individuals that were not claimed by the relatives of the deceased were incorporated into the identified collection, with prior authorization from the respective cemeteries. This study and compilation of the osteological collection was performed in line with the principles of the Declaration of Helsinki and is approved by the Complutense University of Madrid Ethics Committee (Ref: CE_20230209-06_SAL) complying with all the protocols in force for the scientific use of the collection. Sensitive information was anonymized to ensure the protection of personal data.

**Fig. 1:**
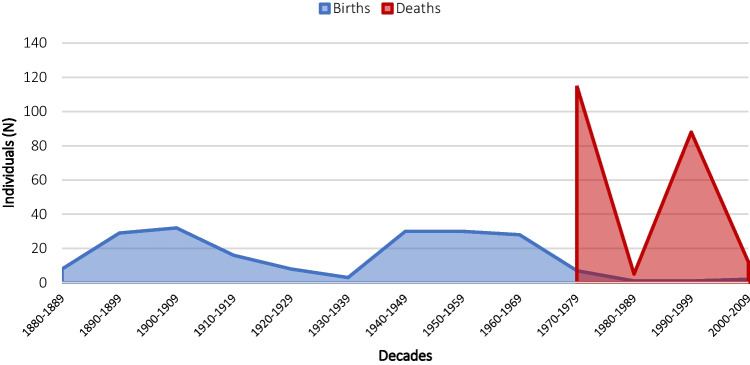
Distribution of decades of birth (blue) and death (red)

The remains come from two cemeteries in the Madrid metropolitan area: 135 individuals come from the Alcorcón Cemetery, which is located about 15 km from the center, and 103 individuals from the South Cemetery of Madrid, which is located in the southeast. Although all the individuals were buried in Madrid, they were born in different Spanish Autonomous Communities and provinces, as the cemeteries’ record shows, and, according to Del Río Muñoz [17], the cranial morphology of the individuals indicates a European ancestry and most of the individuals were born during the 20th century (Fig. 1). For all the reasons mentioned, this sample is considered to be representative of a modern sample, and its population characteristics can be used to develop accurate methods for the current Spanish population.

The cemetery staff informs the laboratory about the dates of the exhumations of the individuals according to the date of burial [24]. They also send an anonymized database where only the biological profile and cause of death are mentioned when known. Cases and ages of interest are selected, and researchers from the Laboratory attend the exhumation of the previously selected individual. When the exact age of death is not known, this data is obtained from the grave information itself.

## Methodology of collecting the skeletal remains

Cemetery workers remove the corpse from the coffin and place it in a clean coffin labeled with an identification code, which is transported to the examination room located in the cemetery. Once transported, the shroud, clothing, and associated objects are removed, and the individual is placed on a work table. The cleaning protocol differs depending on the state of decomposition of the corpse, but generally, all remains of organic matter are carefully cleaned and removed with special attention to teeth and small bones. Once cleaned, the bones are left to dry completely at room temperature. They are then placed in separate plastic bags according to their anatomical region, labeled with a code containing the initials of the cemetery and an identification number. These bags are later stored in cardboard boxes with the code of that individual and transported to the Laboratory of Anthropology and Forensic Odontology at the School of Legal Medicine of the Complutense University of Madrid (UCM).

At the laboratory, technicians receive the boxes for each individual, place them on work tables in anatomical arrangement, and label all the bones with permanent ink and with the identification code of the skeleton. Simultaneously, a bone inventory is developed on a worksheet and then added to each individual’s box. These boxes are stored in a specialized room located in the Department of Legal Medicine of the UCM. All the information obtained is stored in an Excel database to facilitate information gathering for the different lines of research.

## Description of the collection

### Demographic composition

The Identified Skeletal Collection of the School of Legal Medicine consists of 238 individuals of known sex and age, 138 males and 95 females. The mean age at the time of death is 56.01 (SD: 20.974), and the median is 55 years. Table 2 shows the correlation between age and sex in 10-year intervals to facilitate comparison with other published studies. The minimum age of the sample is perinatal, and the maximum age is 97. Table 3 represents the descriptive statistics for age with respect to sex, where it is evident that female individuals are older than male individuals. There are 5 individuals of unknown sex and age accounting for 2.10% of the sample. Figure 1 shows the population distribution in relation to births (between 1889 and 1989) and deaths (between 1970 and 2009).

**Table 2:** Sample distribution by sex and age groups

Age group	Male		Female		Unknown		Total	
	*N*	%	*N*	%	*N*	%	*N*	%
0–4	4	1.7	1	0.4	0	0.0	5	2.1
5–9	1	0.4	0	0.0	0	0.0	1	0.4
10–19	2	0.8	0	0.0	0	0.0	2	0.8
20–29	8	3.4	3	1.3	1	0.4	12	5.0
30–39	26	10.9	11	4.6	0	0.0	37	15.5
40–49	21	8.8	9	3.8	1	0.4	31	13.0
50–59	32	13.4	11	4.6	0	0.0	43	18.1
60–69	12	5.0	13	5.5	0	0.0	25	10.5
70–79	18	7.6	23	9.7	0	0.0	41	17.2
80–89	11	4.6	20	8.4	0	0.0	31	13.0
90+	2	0.8	3	1.3	0	0.0	5	2.1
Unknown	1	0.4	1	0.4	3	1.3	5	2.1
Total	138	58.0	95	39.9	5	2.1	238	100

**Table 3:** Descriptive statistics of the osteological collection

Age	Male	Female	Global
Mean	50.88	63.96	56.01
Median	50	68	55
SD	20.309	19.503	20.974
Min.	0	2	0
Max.	94	97	97

### Cause of death

**Fig. 2:**
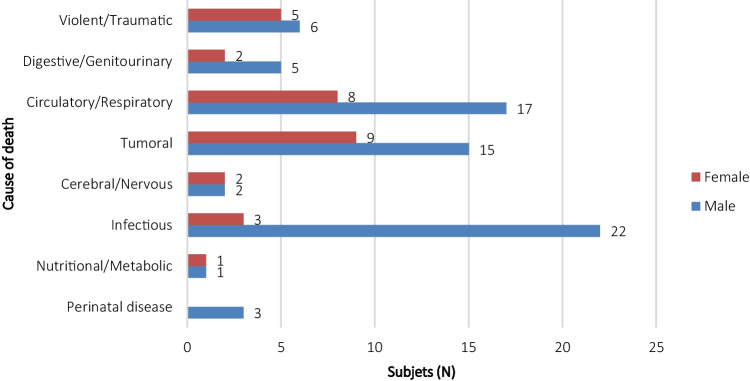
Distribution of cause of death by sex

The cause of death of 103 individuals from the collection is known. 86.40% died due to natural causes, and 10.70% due to violent causes, most of them polytrauma resulting from traffic accidents. 2.90% of the individuals died within hours of birth due to cord prolapse or hypoxia, among others (Table 4). Male individuals (22/103) died from complications due to human immunodeficiency virus (HIV) infection, followed by cardiorespiratory arrest (17/103). Women mainly died from tumors (9/103) and cardiorespiratory arrest (8/103) (Fig. 2).

**Table 4:** Manner of death

	Perinatal		Natural		Violent		Total	
	*N*	%	*N*	%	*N*	%	*N*	%
Male	3	4.20	62	87.30	6	8.50	71	70.30
Female	0	0.00	25	83.30	5	16.70	30	29.70
Total	3	3.00	87	86.10	11	10.90	101	100.00

### State of preservation and completeness

The collection presents different states of preservation depending, above all, on the age of the individual under study, although most are complete and in a very good state of preservation. Even so, older individuals exhibit deterioration of the skeletal remains, which affects the overall quality of the sample. Remains with a higher content of cancellous tissue (e.g. sacrum, vertebrae, and ribs) are in some cases more fragmented. Individuals exhumed 10 years after death can be found in different states of decomposition, from completely skeletonized individuals to challenges with soft putrilage and adipocere, which can affect the state of preservation. In addition, the taphonomic conditions specific to each grave cause different colorations due to the decomposition processes and microclimates present in each grave [25, 26].

## Potential and contribution of the collection

Since its origins, the collection has been instrumental in various projects which have been published in high-impact journals and presented at national and international conferences [20, 25–34]. It has served as a reference collection of a Spanish sample with a good quality of preservation and has served to develop final degree projects, master’s theses, and doctoral dissertations [17, 21, 22, 35]

The anthropological study of the collection is ongoing, and several different lines of research are being carried out, ranging from physical and forensic anthropology to taphonomy and comparative anatomy. Currently, the ontogeny of anatomical variants in the Spanish population [27], differences in muscle markers of activity, the impact of late decomposition in corpses exhumed from the South Cemetery [25, 26], and the mechanism of injury in polytrauma, among other scientific areas, are being studied. In addition, specific methodologies have been developed for a contemporary Spanish population using the clavicle [20, 35], talus [28], mandible [22], long bones [21], and teeth [36, 37] for biological profile estimation.

Access to exhumed bodies allows us to teach students and professional anthropologists about the process of skeletonization, which is essential for understanding the taphonomic and decomposition context of a corpse. Likewise, the studies carried out in this collection have allowed us to improve the biological profile estimation methodologies used in identification cases, which have been subsequently applied in forensic contexts, for historical memory, or in individuals of unknown origin. The agreement with the funeral services of the Community of Madrid is still active, which means that this collection is constantly growing, increasing the sample size and the information available in all lines of research.

## Point of contact

The collection is available for research purposes which may include but are not limited to, population comparisons, biological profile estimations, and validation of methodologies. Interested researchers should submit their proposals and main objectives to the expert committee of the School of Legal Medicine (Forensic Anthropology and Odontology Laboratory at emedicin@ucm.es. Successful applicants will be granted access to the collection.
